# Supplementary material for: Large-scale phenotypic drug screen identifies neuroprotectants in zebrafish and mouse models of retinitis pigmentosa
Source: eLife. 2021 Jun 29;10:e57245. doi: 10.7554/eLife.57245 (PMC8425951; doi:10.7554/eLife.57245)
Supplement: Supplementary file 2. — (a) Compounds tested as positive controls. List of 17 compounds previously reported as neuroprotectants in RP models tested for survival effects in rho:YFP-NTR zebrafish larvae using the primary screening protocol. (b) List of eliminated compounds. Compounds that were autofluorescent (precluding YFP signal detection) or lethal at the concentrations tested (10 mM to 0.625 mM). (c) List of 113 hit compounds. Hit compounds producing a SSMD score ≥1 in the primary screen ordered according to SSMD score. Drug names, concentrations producing SSMD ≥1, SSMD scores, SSMD effect types, and whether a dose-dependent trend was observed or not are shown. Yellow highlighted drugs were selected for confirmation testing. ‘'●” denotes confirmed lead compounds (source data). (d) On-label MOA for 113 hit compounds. Implicated MOA categories and subcategories are listed in order from most common to least common. The number of compounds per each category/subcategory are provided in the parentheses and compound names are listed. [file elife-57245-supp2.docx]

**Supplementary File 2a**

| **Proposed MOA** | **Tested Compounds (abbrv.)** | **References** |
| --- | --- | --- |
| **Antioxidant** | N-Acetyl-L-cysteine (NAC) | (Lee et al., 2011; Schimel et al., 2011) |
|  | N-tert-Butyl-α-(2-sulfophenyl)nitrone | (Mandal et al., 2011) |
| **Hormones and analogs** | 17β-Estradiol | (Nonaka et al., 2000) |
|  | D-(-)-Norgestrel | (Doonan et al., 2011) |
| **PPAR agonist** | Rosiglitazone | (Doonan et al., 2009) |
| **Hsp90 inhibitor** | 17-(Allylamino)-17-demethoxygeldanamycin (17-AAG) | (Tam et al., 2010) |
| **Ceramide synthesis inhibitor** | Myriocin | (Strettoi et al., 2010) |
| **α2-adrenergic agonist** | Clonidine | (Chao and Osborne, 2001) |
| **RTK inhibitor** | Sunitinib | (Latham et al., 2015) |
| **Necrosis inhibitor** | Necrostatin-1 | (Dong et al., 2012) |
| **β-adrenoceptor antagonist** | Levobetaxolol | (Agarwal et al., 2002) |
| **Calpain inhibitor** | Calpastatin peptide | (Paquet-Durand et al., 2010) |
|  | Calpeptin | (Das et al., 2006) |
| **HDAC inhibitor** | Vorinostat (SAHA) | (Berner and Kleinman, 2016; Zhang et al., 2015) |
|  | Romidepsin (FK228) | (Zhang et al., 2015) |
|  | Panobinostat (LBH589) | (Zhang et al., 2015) |
|  | Entinostat (SNDX-275) | (Zhang et al., 2015) |
| **Antioxidant cocktail** | Alpha-tochopherol | (Komeima et al., 2007, 2006) |
|  | Ascorbic acid |  |
|  | Alpha-lipoic acid |  |
|  | MnTBAP |  |

**Compounds tested as positive controls**

List of 17 compounds previously reported as neuroprotectants in RP models tested for survival effects in *rho:YFP-NTR* zebrafish larvae using the primary screening protocol.

**Supplementary File 2b**

| **Autofluorescent Compounds**  **(27)** | Acriflavine (acriflavinium hydrochloride)  Acriflavine hydrochloride  Aklavine hydrochloride  Alexidine hydrochloride  Aromatic cascara fluid extract  Benserazide  Danthron  Dithiazanine iodide (3,3 diethylthiadicarbocyanine iodide)  Fluorescein  Green tea polyphenols  Juniper tar  Merbromin  Nitroxoline (8-hydroxy 5-nitroquinoline)  Nogalamycin  Oftasceine (calcein)  Paroxetine hydrochloride  Phenylmercuric acetate  Proflavine hemisulfate salt hydrate  Protamine chloride  Pyronin Y  Pyrvinium pamoate  Quinalizarin  Riboflavin  Riboflavin tetrabutyrate  Rose Bengal  Rutilantin  Safranin O |
| --- | --- |
| **Lethal Compounds**  **(6)** | Cetylpyridinium (Pyrisept)  Closantel  Hexadimethrine bromide  Iodine  Methyl violet 2B  Polymyxin B sulfate |

**List of eliminated compounds**

Compounds that were autofluorescent (precluding YFP signal detection) or lethal at the concentrations tested (10 mM to 0.625 mM).

**Supplementary File 2c**

| **Leads** | **Drug Name** | **SSMD** | **Conc.** | **Effect**  **subtype** | **Dose-dependent trend** |
| --- | --- | --- | --- | --- | --- |
|  | Aluminum chloride hexahydrate | 2.05;1.16 | 4;2 | Strong | Yes |
|  | Myrrh oil | 1.85 | 4 | Fairly strong | No |
| ● | Miconazole | 1.71; 1.77 | 2; 1 | Fairly strong | No |
|  | Digoxin | 1;1.67 | 4;0.25 | Fairly strong | No |
| ● | Dihydroartemisinin | 1.59 | 4 | Moderate | Yes |
| ● | Ciclopirox olamine | 1.56;1.28;1.05 | 4;2;0.5 | Moderate | Yes |
|  | Isopropamide Iodide | 1.54;1.27 | 2;0.125 | Moderate | No |
|  | Lactulose | 1.52 | 0.25 | Moderate | Yes |
| ● | Warfarin | 1.51;1.05 | 4;2 | Moderate | Yes |
|  | Ethohexadiol | 1.5 | 4 | Moderate | No |
|  | 6,7-Dihydroxyflavone | 1.12;1.04;1.48 | 1;0.25;0.125 | Moderate | No |
|  | Thiostrepton | 1.48 | 4 | Moderate | Yes |
| ● | Cloxyquin | 1.47 | 4 | Moderate | Yes |
|  | Peruvian balsam | 1.46 | 0.5 | Moderate | No |
|  | Almond oil from prunus dulcis | 1.41 | 0.25 | Moderate | No |
|  | Danthron | 1.41 | 0.25 | Moderate | Yes |
|  | 2-Amino-5-(4-nitro-phenylsulfonyl)thiazole | 1; 1.4; 1.06 | 2; 0.5; 0.25 | Moderate | No |
|  | Indomethacin | 1.38 | 0.125 | Moderate | Yes |
|  | Escitalopram oxalate | 1.34 | 0.125 | Moderate | No |
|  | Naproxen sodium | 1.34 | 0.25 | Moderate | No |
|  | Isoxsuprine hydrochloride | 1.31 | 0.25 | Moderate | No |
| ● | Zinc pyrithione | 1.31 | 2 | Moderate | Yes |
|  | Diazepam | 1.3 | 0.125 | Moderate | Yes |
| ● | Calcimycin | 1.3 | 1 | Moderate | Yes |
|  | Amantadine Hydrochloride | 1.28 | 0.25 | Moderate | No |
|  | Nipecotic acid | 1.27 | 0.125 | Fairly moderate | No |
|  | Butoconazole nitrate | 1.27 | 2 | Fairly moderate | No |
|  | Pilocarpine hydrochloride | 1.26 | 2 | Fairly moderate | No |
|  | Hydroquinone | 1.24;1.02 | 2;0.5 | Fairly moderate | No |
|  | Acacetin | 1.12;1.24 | 4;0.25 | Fairly moderate | No |
|  | Eupatorin | 1.24 | 4 | Fairly moderate | Yes |
|  | Neohesperidin dihydrochalcone | 1.24 | 0.125 | Fairly moderate | No |
|  | Pseudoephedrine, (1S,2S)-(+)- | 1.24 | 0.25 | Fairly moderate | Yes |
|  | 5-fluoro-5'-deoxyuridine | 1.22 | 0.25 | Fairly moderate | No |
|  | 2-[N-(3-Phenylpropyl)thiocarbamoyl]-L-cysteine | 1.22 | 4 | Fairly moderate | Yes |
|  | Rathyronine | 1.21 | 0.25 | Fairly moderate | No |
|  | Chlorquinaldol | 1.21 | 4 | Fairly moderate | No |
|  | Streptomycin sulfate | 1.03;1.2 | 4;1 | Fairly moderate | No |
|  | Orthothymotinic acid | 1.19 | 1 | Fairly moderate | No |
|  | Compactin | 1.19 | 2 | Fairly moderate | Yes |
|  | Quinoline | 1.19 | 0.25 | Fairly moderate | No |
|  | Ampicillin sodium salt | 1.19 | 0.25 | Fairly moderate | Yes |
|  | Cyclocreatine | 1.17 | 1 | Fairly moderate | No |
|  | Succimer | 1.17 | 0.5 | Fairly moderate | No |
|  | Rose oil | 1.17 | 0.25 | Fairly moderate | Yes |
|  | Bopindolol maleate | 1.16 | 0.25 | Fairly moderate | No |
|  | Pentolinium | 1.16 | 0.25 | Fairly moderate | Yes |
|  | Oxytetracycline hemicalcium salt | 1.16 | 4 | Fairly moderate | Yes |
|  | Dantrolene sodium | 1.14 | 0.125 | Fairly moderate | No |
|  | Methylatropine nitrate | 1.14;1 | 4;0.5 | Fairly moderate | No |
|  | Magnesium gluconate | 1.14 | 4 | Fairly moderate | Yes |
|  | Leucovorin Calcium | 1.1;1;1.13 | 4;0.5;0.125 | Fairly moderate | No |
|  | Lobeline sulfate | 1.13;1.1 | 0.25;0.125 | Fairly moderate | Yes |
|  | Mercaptamine hydrochloride | 1.12;1.05 | 1;0.5 | Fairly moderate | No |
|  | Chlordiazepoxide | 1.07;1.12;1.11 | 4;1;0.125 | Fairly moderate | No |
|  | Panthenol | 1.12 | 4 | Fairly moderate | No |
|  | 1,2-bis(trimethylsilyloxy)ethane | 1.11 | 0.25 | Fairly moderate | No |
|  | Methyl orange | 1.11 | 4 | Fairly moderate | Yes |
|  | Dyclonine hydrochloride | 1.1 | 0.5 | Fairly moderate | No |
|  | 3-Hydroxybenzylhydrazine dihydrochloride | 1.1;1.07 | 0.25;0.125 | Fairly moderate | Yes |
|  | NCS-382 | 1.1;1.04 | 2;0.5 | Fairly moderate | No |
|  | 1,3 Diethyl 2 thiobarbituric acid | 1.1 | 4 | Fairly moderate | No |
| ● | Cortexolone | 1.1 | 0.125 | Fairly moderate | Yes |
|  | 3-Amino-4-methoxybenzoic acid | 1.1 | 0.125 | Fairly moderate | No |
|  | Hydroxyquinoline benzoate | 1.1 | 2 | Fairly moderate | No |
|  | Iodoquinol | 1.09 | 0.25 | Fairly moderate | No |
|  | Rofecoxib | 1.09 | 0.125 | Fairly moderate | Yes |
| ● | Sulindac | 1.09 | 0.25 | Fairly moderate | Yes |
|  | Terephthalic acid | 1.09 | 4 | Fairly moderate | No |
|  | 4-Hydroxy-6-Methylpyran-2-One | 1.08 | 0.125 | Fairly moderate | No |
|  | D-Galactosamine hydrochloride | 1.08 | 0.25 | Fairly moderate | Yes |
|  | Strophanthin K | 1.08 | 0.125 | Fairly moderate | No |
|  | Lincomycin hydrochloride | 1.07 | 4 | Fairly moderate | No |
|  | Ethaverine hydrochloride | 1.07 | 4 | Fairly moderate | No |
|  | Hydroxypropyl cellulose | 1.07 | 1 | Fairly moderate | No |
|  | Strychnine | 1.06 | 0.25 | Fairly moderate | Yes |
|  | Pyrantel | 1.06 | 0.5 | Fairly moderate | No |
|  | Bamethan sulfate | 1.06 | 1 | Fairly moderate | No |
|  | Deoxycytidylic acid | 1.06 | 0.25 | Fairly moderate | No |
|  | Belladonna tincture | 1.06 | 0.125 | Fairly moderate | Yes |
| ● | Artemisinin | 1.06 | 0.5 | Fairly moderate | No |
|  | Frequentin | 1.05 | 0.25 | Fairly moderate | No |
|  | O-Phenanthroline | 1.05 | 0.5 | Fairly moderate | No |
|  | Hexamethylene glycol | 1.05 | 1 | Fairly moderate | No |
|  | Dichlorisone acetate | 1.05 | 0.25 | Fairly moderate | No |
|  | β-Estradiol 3-benzoate | 1.05 | 0.25 | Fairly moderate | No |
|  | Ellman's reagent | 1.05 | 4 | Fairly moderate | No |
|  | Clopidogrel sulfate | 1.04 | 2 | Fairly moderate | Yes |
|  | Lovastatin | 1.04;1.01 | 0.5;0.25 | Fairly moderate | Yes |
|  | Harmine | 1.04 | 0.25 | Fairly moderate | Yes |
|  | Xanthurenic acid | 1.03 | 0.25 | Fairly moderate | Yes |
|  | Aclarubicin | 1.03 | 4 | Fairly moderate | No |
|  | Menadione | 1.03 | 1 | Fairly moderate | Yes |
|  | Methylphedrine ((1R,2S)-(-)-N-Methylephedrine) | 1.03 | 1 | Fairly moderate | No |
|  | Aluminum lactate | 1.03 | 2 | Fairly moderate | No |
|  | Hexamethonium Chloride | 1.03 | 0.25 | Fairly moderate | No |
|  | Vitamin B4 | 1.03 | 0.25 | Fairly moderate | Yes |
|  | Triethylene glycol | 1.03 | 0.5 | Fairly moderate | No |
|  | Deltaline | 1.02 | 0.25 | Fairly moderate | Yes |
|  | Sennoside A | 1.02 | 0.125 | Fairly moderate | Yes |
| ● | Chloroxine | 1.01 | 4 | Fairly moderate | No |
|  | Tetrabromophenolphthalein ethyl ester, Potassium salt | 1.01 | 0.25 | Fairly moderate | Yes |
|  | Methyl ethyl ketone | 1.01 | 0.125 | Fairly moderate | No |
|  | 3,3,5-Triiodo L-thyronine | 1.01 | 0.5 | Fairly moderate | No |
|  | Nalidixic acid | 1 | 2 | Fairly moderate | Yes |
|  | Putrescine dihydrochloride | 1 | 0.5 | Fairly moderate | No |
|  | Acetazolamide | 1 | 0.25 | Fairly moderate | No |
|  | Betaine | 1 | 4 | Fairly moderate | No |
|  | Maleic acid | 1 | 0.5 | Fairly moderate | No |
|  | Norfenefrine | 1 | 0.5 | Fairly moderate | No |
|  | Azure A | 1 | 0.25 | Fairly moderate | Yes |
|  | Chlorpheniramine | 1 | 0.5 | Fairly moderate | No |
|  | Mustard oil | 1 | 4 | Fairly moderate | Yes |

**List of 113 hit compounds**

Hit compounds producing a SSMD score ≥1 in the primary screen ordered according to SSMD score. Drug names, concentrations producing SSMD ≥1, SSMD scores, SSMD effect types, and whether a dose-dependent trend was observed or not are shown. Yellow highlighted drugs were selected for confirmation testing. “●” denotes confirmed lead compounds (Supplementary File 2c-source data).

**Supplementary File 2d**

| **MOA Category**  **(# cmpds)** | **Subcategory (# cmpds)** | **Compound Name** |
| --- | --- | --- |
| **Neurotransmitter**  **modulator**  **(17)** | GABA signaling (4) | 3-Hydroxybenzylhydrazine dihydrochloride  Chlordiazepoxide  Diazepam  Nipecotic acid |
|  | Cholinergic signaling (3) | Isopropamide Iodide  Methylatropine nitrate  Pilocarpine hydrochloride |
|  | Nicotinic receptor modulator (3) | Hexamethonium chloride  Pentolinium  Pyrantel |
|  | Dopamine release (2) | Lobeline sulfate  Amantadine hydrochloride |
|  | Serotonin reuptake inhibitor (2) | Chlorpheniramine  Escitalopram oxalate |
|  | Glycine/Acetylcholine receptor antagonist (1) | Strychnine |
|  | Glutamate signaling (1) | Xanthurenic acid |
|  | GHB receptor antagonist (1) | NCS-382 |
| **Ion transport**  **modulator**  **(9)** | Sodium channel blocker (2) | Deltaline  Dyclonine hydrochloride |
|  | Na+/K+ ATPase inhibitor (2) | Digoxin  Strophanthin K |
|  | Calcium modulator (2) | Dantrolene sodium  Ethaverine hydrochloride |
|  | Multiple ions carrier (2) | Calcimycin  Succimer |
|  | Proton (1) | Zinc pyrithione |
| **Adrenergic receptor modulator**  **(6)** | Beta-adrenergic modulator (3) | Bamethan sulfate  Bopindolol maleate  Isoxsuprine hydrochloride |
|  | α and β receptors modulator (3) | Methylphedrine ((1R,2S)-(-)-N-Methylephedrine)  Norfenefrine  Pseudoephedrine, (1S,2S)-(+)- |
| **Antibacterial**  **agent**  **(6)** | Protein synthesis inhibitor (4) | Lincomycin hydrochloride  Oxytetracycline hemicalcium salt  Streptomycin sulfate  Thiostrepton |
|  | Peptidoglycan synthesis inhibitor (1) | Ampicillin sodium salt |
|  | Unknown (1) | Aluminum lactate |
| **Hormone**  **related**  **(5)** | Thyroid hormone (2) | 3,3,5-Triiodo L-thyronine  Rathyronine |
|  | Cortecosteroid (2) | Cortexolone  Dichlorisone acetate |
|  | Estrogen (1) | β-Estradiol 3-benzoate |
| **Chelating agent (5)** | | Cloxyquin  Hydroxyquinoline benzoate  Iodoquinol  O-Phenanthroline  Quinoline |
| **Therapeutic plant extract (5)** | | Almond oil from prunus dulcis  Belladonna tincture  Mustard oil  Myrrh oil  Rose oil |
| **NSAID (4)** | COX1 inhibitor (2) | Indomethacin  Sulindac |
|  | COX2 inhibitor (1) | Rofecoxib |
|  | Nonselective COX inhibitor (1) | Naproxen sodium |
| **Antioxidant (4)** | Flavonoid (3) | 6,7-Dihydroxyflavone  Acacetin  Eupatorin |
|  | Glutathione S-transferase inducer (1) | 2-[N-(3-Phenylpropyl)thiocarbamoyl]-L-cysteine |
| **Vitamin (4)** | | Vitamin B4  Panthenol  Menadione  Leucovorin calcium |
| **DNA synthesis inhibitor and cleavage (3)** | | Nalidixic acid; 5-Fluoro-5'-Deoxyuridine; Aclarubicin |
| **Antifungal agent (3)** | Ergosterol inhibition(2) | Butoconazole nitrate  Miconazole |
|  | Catalase and endoperoxide enzyme inhibitor (1) | Ciclopirox olamine |
| **Antimalarial (2)** | | Artemesinin  Dihydroartemisinin |
| **Antimicrobial agent (2)** | | Chloroxine  Chlorquinaldol |
| **Anticoagulant (2)** | | Clopidogrel sulfate  Warfarin |
| **HMG-CoA reductase inhibitor (2)** | | Compactin  Lovastatin |
| **MAO-A inhibitor (2)** | | Harmine  Sennoside A |
| **Others (19)** | | 1,3 Diethyl 2 thiobarbituric acid  Acetazolamide  Aluminum chloride hexahydrate  Azure A  Betaine  Cyclocreatine  D-Galactosamine hydrochloride  Danthron  Deoxycytidylic acid  Ellman's reagent  Ethohexadiol  Hexamethylene glycol (1,6 hexane diol)  Hydroquinone  Hydroxypropyl cellulose  Lactulose  Magnesium gluconate  Maleic acid  Mercaptamine Hydrochloride  Methyl orange |
| **Unknown (13)** | | 1,2-bis(trimethylsilyloxy)ethane  2-Amino-5-(4-nitro-phenylsulfonyl)thiazole  3-Amino-4-methoxybenzoic acid  4-Hydroxy-6-Methylpyran-2-One  Frequentin  Methyl ethyl ketone (2-Butanone)Neohesperidin dihydrochalcone  Orthothymotinic acid  Peruvian balsam  Putrescine dihydrochloride  Terephthalic acid  Tetrabromophenolphthalein ethyl ester, Potassium salt  Triethylene glycol |

**On-label MOA for 113 hit compounds**

Implicated MOA categories and subcategories are listed in order from most common to least common. The number of compounds per each category/subcategory are provided in the parentheses and compound names are listed.
