# Supplementary material for: Large-scale phenotypic drug screen identifies neuroprotectants in zebrafish and mouse models of retinitis pigmentosa
Source: eLife. 2021 Jun 29;10:e57245. doi: 10.7554/eLife.57245 (PMC8425951; doi:10.7554/eLife.57245)
Supplement: Supplementary file 3. — Abbreviations: parp1, poly (ADP-ribose) polymerase 1; ripk1l, receptor (TNFRSF)-interacting serine-threonine kinase 1, like; casp3a: caspase 3, apoptosis-related cysteine peptidase a; casp3b: caspase 3, apoptosis-related cysteine peptidase b; tdp1, tyrosyl-DNA phosphodiesterase 1; actb1: actin, beta 1; rplp0: ribosomal protein, large, P0. [file elife-57245-supp3.docx]

**Supplementary File 3**

| **Names** | **Oligonucleotides used for synthesizing sgRNA** |
| --- | --- |
| *parp1*_Oligo_1 | TAATACGACTCACTATAGGTAGCACCTCCACTCTCAAGTTTTAGAGCTAGAAATAGC |
| *parp1*_Oligo_2 | TAATACGACTCACTATAGGAGCGGGAGGAGCGTCTTTGTTTTAGAGCTAGAAATAGC |
| *parp1*_Oligo_3 | TAATACGACTCACTATAGGTCGAGTCGGGACCACCATGTTTTAGAGCTAGAAATAGC |
| *parp1*_Oligo_4 | TAATACGACTCACTATAGGCTTCATGCCGAAGTCGTGGTTTTAGAGCTAGAAATAGC |
| *ripk1l*_Oligo_1 | TAATACGACTCACTATAGGCACCAGGACCCTCTGCAGGTTTTAGAGCTAGAAATAGC |
| *ripk1l_*Oligo*_2* | TAATACGACTCACTATAGGCCGGGACGCTGTGCTACAGTTTTAGAGCTAGAAATAGC |
| *ripk1l*_Oligo_3 | TAATACGACTCACTATAGGAGGATGGAGATTATTCATGTTTTAGAGCTAGAAATAGC |
| *ripk1l*_Oligo_4 | TAATACGACTCACTATAGGAGGAGCTGTATCCCAGAGGTTTTAGAGCTAGAAATAGC |
| *casp3a*_Oligo_1 | TAATACGACTCACTATAGGCTTGGCATCAACCTGCATGTTTTAGAGCTAGAAATAGC |
| *casp3a*_Oligo_2 | TAATACGACTCACTATAGGTACGTCAGTGCCATTGCGGTTTTAGAGCTAGAAATAGC |
| *casp3a*_Oligo_3 | TAATACGACTCACTATAGGATAATCTGCGCAACTGTCGTTTTAGAGCTAGAAATAGC |
| *casp3a*_Oligo_4 | TAATACGACTCACTATAGGTCAGTCACGGCGATGAGGGTTTTAGAGCTAGAAATAGC |
| *casp3b*_Oligo_1 | TAATACGACTCACTATAGGATCCATCTGAACGAGTCCGTTTTAGAGCTAGAAATAGC |
| *casp3b*_Oligo_2 | TAATACGACTCACTATAGGATCATCTGTGAGACGGTCGTTTTAGAGCTAGAAATAGC |
| *casp3b*_Oligo_3 | TAATACGACTCACTATAGGAGACGACGGGCTGATCTAGTTTTAGAGCTAGAAATAGC |
| *casp3b*_Oligo_4 | TAATACGACTCACTATAGGGTTTCTCCAGGCCTGCCGGTTTTAGAGCTAGAAATAGC |
| *tdp1*_Oligo_1 | TAATACGACTCACTATAGGAGCATCCGCTCCCTCCATGTTTTAGAGCTAGAAATAGC |
| *tdp1*_Oligo_2 | TAATACGACTCACTATAGGATCCGCCAGAATTTAGGTGTTTTAGAGCTAGAAATAGC |
| *tdp1*_Oligo_3 | TAATACGACTCACTATAGGGCTCCAGAGCTCGCCAACGTTTTAGAGCTAGAAATAGC |
| *tdp1*_Oligo_4 | TAATACGACTCACTATAGGAGAGCGATGGCACGTGCTGTTTTAGAGCTAGAAATAGC |
| scaffold oligo | AAAAGCACCGACTCGGTGCCACTTTTTCAAGTTGATAACGGACTAGCCTTATTTTAACTTGCTATTTCTAGCTCTAAAAC |
| **Names** | **qPCR primers** |
| *parp1*-F | CAGACCCAAGAGCTACAGTATC |
| *parp1*-R | GGATCTTCAGCAGGTATTTCAGG |
| *ripk1l*-F | ACCACCAGCAGATACAAAGAG |
| *ripk1l*-R | TTCGCTCAGACCCAGTTTAC |
| *casp3a*-F | CCAGATGGTCGTGTAAGGATC |
| *casp3a*-R | GTTCACTGCCATACTTTGTCATC |
| *casp3b*-F | CATTGAGTGTGACGGTGTAGG |
| *casp3b*-R | GGAGATAAACCAGGAGCCATTAG |
| *tdp1*-F | AGAATGTGTTTCCGGCCTG |
| *tdp1*-R | TGTCTGGTGCTTGAGTGTATG |
| *actb1*-F | ATCTTCACTCCCCTTGTTCAC |
| *actb1*-R | TCATCTCCAGCAAAACCGG |
| *rplp0-*F | CAACCTTGTCTTTAAACCGGC |
| *rplp0*-R | GCCCACGATGAAACACTTG |

**Oligonucleotides used for sgRNA synthesis (gene knockdown) and qPCR primers**.

Abbreviations: *parp1*, *poly (ADP-ribose) polymerase 1*; *ripk1l*, *receptor (TNFRSF)-interacting serine-threonine kinase 1, like*; *casp3a*: *caspase 3, apoptosis-related cysteine peptidase a*; *casp3b*: *caspase 3, apoptosis-related cysteine peptidase b*; *tdp1*, *tyrosyl-DNA phosphodiesterase 1*; *actb1*: *actin, beta 1*; *rplp0*: *ribosomal protein, large, P0*.
